# Impact of fecal microbiota transplantation on chronic recurrent pouchitis in ulcerative colitis with ileo-anal anastomosis: study protocol for a prospective, multicenter, double-blind, randomized, controlled trial

**DOI:** 10.1186/s13063-020-04330-1

**Published:** 2020-06-03

**Authors:** Caroline Trang-Poisson, Elise Kerdreux, Alexandra Poinas, Lucie Planche, Harry Sokol, Pascale Bemer, Karine Cabanas, Eliane Hivernaud, Laetitia Biron, Laurent Flet, Emmanuel Montassier, Ghislaine Le Garcasson, Anne Chiffoleau, Alexandra Jobert, Didier Lepelletier, Jocelyne Caillon, Patrice Le Pape, Berthe-Marie Imbert, Arnaud Bourreille

**Affiliations:** 1 Gastroenterology Department, Institute of Digestive Diseases (Institut des Maladies de l’Appareil Digestif - IMAD), CHU Nantes and Nantes University, Nantes, France; 2 Clinical Investigation Centre CIC1413 team IMAD, CHU Nantes and Inserm, Nantes, France; 3 Clinical Investigation Centre CIC1413, CHU Nantes and INSERM, Nantes, France; 4 Methodology and Biostatistics Unit, Delegation to Clinical Research and Innovation for CHU Nantes and Vendée departmental Hospital, Nantes, La Roche sur Yon France; 5 Sorbonne Université, Inserm, Centre de Recherche Saint-Antoine, CRSA, AP-HP, Hôpital Saint Antoine, Gastroenterology & Nutrition Department, F-75012 Paris, France; 6 MiHAR lab, Nantes University, 44000 Nantes, France; 7 Department of Emergency Medicine, CHU Nantes, Nantes, France; 8 Sponsor Department, CHU Nantes, Nantes, France; 9 Pharmacy Department, CHU Nantes, Nantes, France; 10 Bacteriology and Infection Control Department, CHU Nantes, 44000 Nantes, France; 11 Parasitology-Mycology Department, Institute of Biology CHU Nantes, Nantes, France; 12 Virology Department, CHU Nantes, Nantes, France

**Keywords:** Fecal microbiota transplantation, Ileal pouch–anal anastomosis, Pouchitis, Randomized controlled trials

## Abstract

**Background:**

Almost 15% of patients with ulcerative colitis (UC) will require a proctocolectomy with ileal pouch–anal anastomosis (IPAA) as a result of fulminant colitis, dysplasia, cancer, or medical refractory diseases. Around 50% will experience pouchitis, an idiopathic inflammatory condition involving the ileal reservoir, responsible for digestive symptoms, deterioration in quality of life, and disability. Though the majority of initial cases of pouchitis are easily managed with a short course of antibiotics, in about 10% of cases, inflammation of the pouch becomes chronic with very few treatments available.

Previous studies have suggested that manipulating the composition of intestinal flora through antibiotics, probiotics, and prebiotics achieved significant results for treating acute episodes of UC-associated pouchitis. However, there is currently no established effective treatment for chronic antibiotic-dependent pouchitis. Fecal microbiota transplantation (FMT) is a novel therapy involving the transfer of normal intestinal flora from a healthy donor to a patient with a medical condition potentially caused by the disrupted homeostasis of intestinal microbiota or dysbiosis.

**Methods:**

Our project aims to compare the delay of relapse of chronic recurrent pouchitis after FMT versus sham transplantation. Forty-two patients with active recurrent pouchitis after having undergone an IPAA for UC will be enrolled at 12 French centers. The patients who respond to antibiotherapy will be randomized at a ratio of 1:1 to receive either FMT or sham transplantation.

**Discussion:**

On April 30, 2014, the World Health Organization published an alarming report on antibiotic resistance. Finding an alternative medical treatment to antibiotics in order to prevent relapses of pouchitis is therefore becoming increasingly important given the risk posed by multiresistant bacteria. Moreover, if the results of this study are conclusive, FMT, which is less expensive than biologics, could become a routine treatment in the future.

**Trial registration:**

ClinicalTrials.gov, NCT03524352. Registered on 14 May 2018.

## Background

As many as 10%–20% of patients with ulcerative colitis (UC; incidence of 24 per 100,000 people annually in Europe) undergo proctocolectomy with ileal pouch–anal anastomosis (IPAA) as a result of refractory disease, dysplasia, or malignancies [1].

Moreover, more than 50% of those who have had an IPAA for a complication brought on by UC will develop an inflammation of the neo-reservoir, known as pouchitis, in the following years. Pouchitis worsens the quality of life, causing disability, an increase in bowel movements, an increased risk of fecal incontinence, abdominal pains, and fever.

The etiology of pouchitis remains unclear. Risk factors, genetic associations, and the serological markers of pouchitis suggest that a close interaction between the host immune response and the pouch microbiota plays a relevant role in the etiology of this idiopathic inflammatory condition [2–4].

While it is known that both host genetics and the microbiome influence the development of pouchitis, precisely how they interact is less well understood. After IPAA surgery, the mucosal structure of the J-pouch becomes more colon-like: villous structures become shallower, mucin expression changes, and the microbial community becomes functionally more similar to a colonic community. Although pouchitis can occur after the construction of pouches for either chronic ulcerative colitis or familial adenomatous polyposis, pouchitis occurs much less frequently in the latter (0%–10%) [5–7], suggesting that pouchitis is less related to the structure of the pouch than it is the patient’s underlying immune dysregulation and the microbiota interacting with the pouch machiels [8]. It is unclear, however, whether pouchitis is a recurrence of UC that manifests itself as the postoperative host ileum and microbiome collectively become more colon-like, or a unique disease with characteristics of both Crohn’s disease (CD) and UC [9, 10].

Inflammatory bowel disease (IBD) has no single etiology, but is rather the consequence of the detrimental interaction between intestinal microbiota, epithelium, and the immune system in genetically susceptible individuals. The use of modern molecular methods that allow a comprehensive analysis of the gut microbiota has shown that patients with IBD have a distorted, low-diversity intestinal microbiota [11, 12]. There is an ongoing debate as to whether changes in the intestinal microbiota precede or follow the development of colitis in IBD. A specific IBD microbiota has, however, yet to be revealed.

Dysbiosis has been indicated as a triggering factor for pouchitis, and this is strongly supported by several lines of evidence pointing to the role played by the microbiota. Pouchitis does not occur before ileostomy take-down and the ileal mucosa’s exposure to the fecal stream. In addition, the pouch effluent contains higher bacterial concentrations than the normal ileum and studies have reported alterations in bacterial colonization after IPAA.

Treatment of pouchitis is largely empirical and only small placebo-controlled trials have been conducted [13, 14]. Antibiotics are the main treatment for acute pouchitis, and metronidazole and ciprofloxacin are the most common initial approaches, often resulting in a rapid response, suggesting that dysbiosis is indeed involved in this pathology [2]. Treatments such as antibiotics and probiotics have a positive effect on the induction and maintenance of remission in pouchitis [15, 16].

While antibiotics are often effective, 5%–15% of patients experience “refractory or recurrent” pouchitis. Active pouchitis may then be divided into acute or chronic, depending on the symptom duration according to ECCO guidelines [13]. The threshold for chronicity is a symptom duration of > 4 weeks. Up to 10% of patients develop chronic pouchitis requiring long-term treatment and a small subgroup have pouchitis refractory to medical treatment. Pouchitis may be classified—according to different perspectives—into: (1) idiopathic versus secondary; (2) in remission versus active; and (3) infrequent (< 3 episodes/year) versus relapsing (> 3 episodes/year). Pouchitis may also be classified based on the response to antibiotic therapy: (1) antibiotic-responsive; (2) antibiotic-dependent (need for continuous antibiotic treatment to maintain remission); and (3) antibiotic-refractory.

Pouchitis recurs in > 50% of patients. Patients with recurrent pouchitis can be broadly grouped into three categories: infrequent episodes (< 1/year); a relapsing course (1–3 episodes/year); or a continuous course. Pouchitis may further be termed treatment responsive or refractory, based on response to antibiotic monotherapy. Although these distinctions are largely arbitrary, they help both patients and physicians when considering management options to alter the pattern of pouchitis [13].

Hence, the definition of our study population as patients in remission on long-term antibiotics has been made according to this ECCO definition.

Note that antibiotics are significantly associated with a high risk of side effects and the risk of multi-resistant bacteria emerging [17].

Recurrent pouchitis or refractory pouchitis is responsible for deterioration in patients’ quality of life—which has already been altered by the surgery itself—increased disability, and in some cases, unfortunately, the need for immunosuppressive medication to avoid further complications with the ileal pouch.

Probiotics (VSL#3®, 450 billion bacteria of eight different strains per gram) are the only treatment to have been found effective in preventing relapse and maintaining remission in patients with chronic pouchitis [18–20].

Probiotics have anti-inflammatory effects and appear to regulate the mucosal immune response through reductions in pro-inflammatory cytokines. Restoring the integrity of the “protective” intestinal mucosa-related microbiota could therefore be one mechanism by which probiotic bacteria function. However, probiotics are not available in all countries and, as they are not considered medication, their cost is not reimbursed. In addition to which, > 50% of patients are not responders. The crucial lack of treatments for these patients explains the need to develop new therapeutic strategies.

The complete substitution of a dysbiotic intestinal microbiota for a “healthy” one is a promising strategy in the treatment of chronic pouchitis. Indeed, fecal microbiota transplantation (FMT), also known as stool transplantation [21], has been shown to be a successful therapeutic approach in other dysbiosis conditions, such as recurrent *Clostridium difficile* infection (CDI), with an overall resolution rate of around 90% and no safety issues in a recent randomized clinical trial [22]. Although the mechanism of efficacy of FMT on CDI is still unclear, FMT is now internationally recommended in cases of recurrent CDI [23]. The only adverse reactions (ARs) reported until 2019 were transitional bowel discomfort, fever, and abdominal pain after 3 years of FMT [24]. Recently, the Food and Drug Administration (FDA) reported two cases of drug-resistant *E. coli* bacterelia transmitted by FMT with one leading to death in two different clinical trials [25]. Both patients were treated with the same donor. It appears that since November 2018 the routine tests for extended spectrum β-lactamase (ESBL)-producing organisms in donor-screening protocol is mandatory.

Given the evidence of the involvement of dysbiosis in the pathogenesis, interest in the use of FMT to treat IBD is growing. In 2017, Paramsothy et al. conducted a meta-analysis of 53 studies (41 on UC, 11 on CD, and four on pouchitis) [26]. Overall, 36% (201/555) of patients with UC, 50.5% (42/83) of those with CD, and 21.5% (5/23) of those with pouchitis achieved clinical remission. Among cohort studies, the pooled proportion achieving clinical remission was 33% (95% confidence interval [CI] = 23–43) for UC and 52% (95% CI = 31–72) for CD, both with a moderate risk of heterogeneity. For four randomized clinical trials (RCTs) in UC, a significant benefit in clinical remission (pooled odds ratio [P-OR] = 2.89, 95% CI = 1.36–6.13, *p* = 0.006) with moderate heterogeneity (Cochran’s Q, *p* = 0.188; I2 = 37%) was noted [27, 28]. A recent Cochrane analysis including either exclusively randomized trials, or non-randomized studies with a control arm, concluded that fecal microbiota transplantation may increase the proportion of participants with UC achieving clinical remission and that there is a need for additional high-quality studies to further define the optimal parameters of FMT [29].

Only nine publications (seven articles and two abstracts) describing two case reports and two open label studies, concerning a total of 62 patients, have been published on the subject of FMT in chronic pouchitis [30–37]. All of them produced heterogeneous results and these studies alone are clearly insufficient to promote this technique. Furthermore, they concerned the treatment of active pouchitis exclusively excepted one study [35] concerning six patients only. There are currently very few available data concerning the efficacy of FMT in the prevention of chronic recurrent antibiotic-dependent pouchitis. Moreover, only 15 RCTs have been published on pouchitis with five studies on chronic pouchitis, including only two on maintaining remission, both using probiotic therapies [14]. All the published studies use a variety of different designs and methods of evaluation and scores, and there is currently a need for additional high-quality trials of pouchitis treatments, especially novel therapies. As already said, the goal of their trials is the remission of active pouchitis. For the first time, the present study will focus on patients in remission to increase this delay, this free interval without treatment. Moreover, the methodology of previous trials is biased, combining refractory and antibiotic-responsive patients [16, 18, 19, 34–36].

Finally, although seven studies are listed on https://clinicaltrials.gov, only one RCT about the use of FMT to treat pouchitis has yet been published [35].

**Table:** 

**Strengths of this study**	
➢ This multicenter study is one of the first randomized placebo-controlled trials assessing the feasibility and clinical efficacy of FMT in the prevention of recurrences of chronic antibiotic-dependent pouchitis.	
➢ This trial is also a double-blind study: all the syringes containing either donor feces in sterile saline and pharmaceutical glycerol, or sterile saline and pharmaceutical glycerol (10%), will be identical and opaque to ensure blinded conditions.	
➢ Patients’ reported quality-of-life measures will be recorded.	
➢ The present study has a long follow-up period (2 years), allowing the patient to be properly monitored.	
➢ The results of the present study could provide new guidelines for the treatment of chronic recurrent pouchitis.	

Finding an alternative treatment to immunosuppressive therapy in recurrent pouchitis is important for patients in order to improve their quality of life, reduce disability, and avoid complications affecting the pouch. Therefore, the aim of the present study was to examine the benefits of FMT in recurrent pouchitis in terms of its ability to prevent relapse and restore the intestinal microbiota. The present study could lead to future non-immunosuppressive treatments for chronic recurrent pouchitis with the current promising development of encapsulated frozen stool.

## Methods/Design

### Study design

This is a prospective, phase III, randomized, double-blind, placebo-controlled, multicenter, national study to evaluate the use of FMT in preventing relapses of pouchitis in patients with UC who have undergone IPAA.

Approximately 50 patients with active recurrent pouchitis after IPAA for UC will be enrolled at 12 expert French centers, with an average of four patients per center. Forty-two of these individuals who have responded after 4 weeks of antibiotherapy will be randomized to receive FMT or sham transplantation at a ratio of 1:1.

### Study population

#### Patients

As mentioned above, no data have been published on the maintenance in remission after FMT in recurrent pouchitis.

There are approximately 80,000 patients with UC in France (EPIMAD register), and as many as 10%–20% will undergo proctocolectomy with IPAA as a result of refractory disease, dysplasia, or malignancies. Approximately 50% of people who have had an IPAA will develop pouchitis in the following years. Most of these patients are monitored in general or university hospitals.

We anticipate that 80% of the patients with chronic recurrent pouchitis will respond to antibiotherapy, with 5% of loss of view. We estimate that each center will include one patient per year (= 0.08 patient/month).

During the screening visit, the study will be fully explained to the patient and informed consent will be obtained if the patient satisfies all inclusion and non-inclusion criteria (Table 1). When giving informed consent, patients will also be asked for permission for the research team to share relevant data with people from the universities taking part in the research or from regulatory authorities, where relevant (the French version of the informed consent is available from the corresponding author on request).

**Table 1:** Participant selection criteria

**Inclusion criteria**	
Individuals must satisfy the following criteria to be enrolled in the study:	
1. Male or female aged ≥ 18 years at the time of signing the ICF.	
2. Individual must understand and voluntarily sign an ICF before conducting the study-related assessments/procedure.	
3. Willing and able to adhere to the scheduled study visits and other protocol requirements.	
4. Individuals must have been operated with IPAA with a duration of at least 6 month before the screening visit.	
5. Individuals must have a diagnosis of recurrent pouchitis defined as at least two episodes in the last year or relapsing immediately after a reasonable response to antibiotherapy (the antifungal medication is allowed until the day before transplantation).	
6. Individuals must be in remission with a PDAI < 7 at screening	
7. Individuals must have affiliation with a social security system or be a beneficiary of such a system	
8. Women of childbearing age must have a negative pregnancy test at screening and must agree to practice effective methods of contraception	
**Exclusion criteria**	
Individuals who meet any of the following exclusion criteria cannot be enrolled in this study:	
1. Crohn’s disease or indeterminate colitis	
2. Anastomotic stenosis	
3. Individuals with prior treatment by probiotic within 3 months before the transplantation visit	
4. Individuals with prior treatment by corticosteroids within 6 weeks before the transplantation visit	
5. Individuals with prior treatment by immunosuppressors within 3 month before the transplantation visit	
6. Prior treatment with a biologic within 3 month before the transplantation visit	
7. Documented active infection of any kind in the last 6 months	
8. ANC < 1.5 × 10^9^ /L (1500 mm^3^)	
9. Infection with chronic HIV	
10. Pregnant or breastfeeding women	
11. Chronic medical or psychiatric disease that may interfere with the individual’s ability to comply with study procedures	
12. Administration of investigational drugs within 3 months before planned FMT	
13. Adults under guardianship, safeguard justice, or trusteeship	
14. Individuals with difficulty in follow-up (vacation, job transfer, geographical distance, lack of motivation)	
15. Patients with contraindication to colonoscopy or anesthesia (if necessary)	

*ANC* absolute neutrophil count, *FMT* fecal microbiota transplantation, *HIV* human immunodeficiency virus, *ICF* informed consent form, *IPAA* ileal pouch–anal anastomosis, *PDAI* Pouchitis Disease Activity Index

It should be noted that the following drugs, which may interfere with the FMT, are prohibited:

- Antibiotics after transplantation;
- Probiotics in the 3 months before transplantation and for the duration of the study;
- Immunosuppressive therapy including chemotherapy in the 3 months before transplantation visit;
- Biologic drugs are prohibited within the 3 months before transplantation visit;
- Corticosteroids in the 6 weeks before transplantation visit;
- Nonsteroidal anti-inflammatory drugs (NSAIDs) for the duration of the study.

#### Donors

The University Hospital of Nantes will collaborate with public or private organizations to recruit voluntary donors through public announcements or notices after asking previous donors as part of the care for the treatment of *C. difficile* colitis.

We expect about 100 potential donors to give consent, in order to include 12 donors responding to the selection criteria of the ANSM (French regulatory authority) and considering those lost to follow-up. It should be noted that donors will receive a compensation fee.

It should be noted that as a result of the COVID-19 pandemic, an amendment of the protocol will be made to test the donor and patient for the SARS-CoV-2 virus.

### Study schedule

When including donors in the trial, during the screening visit, the study will be fully explained to the donor and informed consent will be obtained. In the informed consent, the donor will have to give permission (ditto for the patient) to share relevant data with people from the universities taking part in the research or from regulatory authorities and to collect biological specimen for use and storage.

The donors must first meet the criteria of the preselection questionnaire to exclude any contraindications. This preselection questionnaire focuses on digestive disorders, drug treatment, and travel. The donor must then undergo a physical examination by a physician, including examination of the anal margin to search for lesions attributable to human papillomavirus (HPV) or herpes simplex virus (HSV). Blood and fecal testing will then be performed, as these tests are required by the ANSM’s dossier on FMT and its supervision in clinical trials [38] (Table 2). It should be noted that for cytomegalovirus (CMV), Epstein–Barr virus (EBV), and *Toxoplasma gondii*, a sero-concordance with the receiver will be performed.

**Table 2:** Infectious disease screening of donors

**Blood test**	
Virology	➢ Human immunodeficiency virus (HIV) ➢ Hepatitis B virus (HBV) ➢ Hepatitis C virus (HCV) ➢ Cytomegalovirus (CMV)^a^ ➢ Epstein–Barr virus (EBV)^a^ ➢ Human T-lymphotopic virus (HTLV)
Bacteriology	➢ Trepenema pallidium
Parasitology	➢ *Strongyloides stercoralis* ➢ *Toxoplasma gondii*^a^ ➢ *Trichinella spiralis* ➢ Amibiasis
**Stool test**	
Virology	➢ Adenovirus ➢ Astrovirus ➢ Calcovirus (norovirus, sapovirus) ➢ Picornavirus (enterovirus, Aichi virus) ➢ Rotavirus ➢ Hepatitis E virus (HEV) ➢ Hepatitis A virus (HAV)
Bacteriology	➢ *Clostridium difficile* ➢ *Listeria monocytogenes* ➢ *Vibrio cholera / Vibrio para-hemolyticus* ➢ Salmonella ➢ Shigella ➢ Yersinia ➢ Campylobacter ➢ Multiresistant bacteria to antibiotics
Parasitology	➢ Strongyloïdes stercoralis ➢ Cryptosporidium sp. ➢ Cyclospora sp. ➢ *Entamoeba histolytica* ➢ Giardia intestinalis ➢ Isospora sp. ➢ Microsporidies ➢ Blastocystis hominis ➢ *Dientamoeba fragilis*

^a^ Sero-concordance with the receiver

The main inclusion criteria for the donors are: adults of both sexes (aged 18–65 years) with a body mass index of 17–30 kg/m^2^ and a usually regular transit of 1–3 stools per day corresponding to types 2–4 on the Bristol scale.

The non-inclusion criteria for the donors are divided into three classes relating to the patient’s status, previous or concomitant treatment, and infectious risk, and these are provided in Tables 2 and 3.

**Table 3:** Exclusion criteria for the donor

**1 Exclusion criteria related to general safety**	
*Personal status*	
*General*	➢ Presence of chronic disease ➢ Pregnant or lactating women ➢ Adults under guardianship, safeguard justice, or trusteeship ➢ For donors over the age of 50 years, absence of screening test for colorectal cancer within 2 years and positive test result
*Digestive*	➢ Celiac disease ➢ Irritable bowel syndrome ➢ Chronic constipation ➢ Diarrhea defined as > 3 very soft or liquid stools per day ➢ Hemorrhoids or rectal bleeding ➢ Personal history of: • gastrointestinal neoplasia or polyps • autoimmune or inflammatory disease • IBD
*Diet and drug use*	➢ Particular diet (exclusion diet, vegetarian diet) or other specific considerations: ingestion of a potential allergen (e.g. peanut) to which the recipient has a known allergy ➢ → Alcohol or drug abuse ➢ Spouse of a patient with IBD
*Family status*	➢ First-degree family history of: • IBD • gastrointestinal neoplasia or polyps before the age of 60 years • autoimmune or inflammatory disease ➢ Diarrhea defined as > 3 very soft or liquid stools per day for the family members within 3 months.
**2 Exclusion criteria related to prior or concomitant treatment**	
	➢ No medication whatsoever in the 48 h before the donation (except contraceptive treatment) ➢ No regular curative medication except oral contraception ➢ Immunosuppressants (e.g. calcineurin inhibitors, corticosteroids, biological agents, etc.) antineoplastic chemotherapy ➢ Antibiotic or antifungal in the previous 3 months before the donation ➢ Non-steroidal anti-inflammatory intake in the previous month for the donation
**3 Exclusion criteria related to infection risk**	
*General*	➢ Abnormal macroscopic appearance of stool ➢ Known infection by HIV, HTLV virus, hepatitis B or C virus ➢ Positive result in one of the contagious disease testing (see Appendix ANSM) ➢ Existence of anal lesions suggestive of viral infection (papillomas, vesicles, or other lesions) ➢ Multiresistant bacteria carrier (see Appendix ANSM) ➢ Risk of Creutzfeldt–Jakob disease ➢ Personal history of typhoid fever
*2 years*	➢ Residence in the intertropical zone > 2 years
*12 months*	➢ Hospitalization abroad > 24 h in the last 12 months (including members of the family)
*6 months*	➢ Contact with human blood (blood transfusion, piercing, tattoo, etc.) within the previous 6 months ➢ Sexual behavior risk (defined as unprotected sexual contact with a new partner) in the previous 6 months
*3 months*	➢ Gastroenteritis in the last 3 months Digestive disorders such as acute or chronic diarrhea in the 3 months preceding the donation ➢ Behavior deemed at risk of infection: travel in the previous 3 months
*14 days*	➢ Infectious episode 14 days before screening
**Blood tests**	
*Biology*	➢ Abnormal local lab value concerning the following tests: fasting glucose, blood count, platelets, ferritin, CRP, ionogram, urea, creatinine, AST/ALT, gamma glutamyl transferase, alkaline phosphatase, bilirubin, prothrombin time, activated partial thromboplastin time, lipids (cholesterol, triglycerides)
*Virology, bacteriology, and parasitology*	See Table 2
**Stool test**	
*Virology, bacteriology, and parasitology*	See Table 2

*CRP* C-reactive protein, *HIV* human immunodeficiency virus; *IBD* Irritable Bowel Syndrome

As with patients, certain treatments are prohibited to donors before fecal donation:

- Antibiotic or antifungal in the 3 months preceding the donation;
- NSAIDs in the month preceding the donation;
- Immunosuppressive drugs (e.g. calcineurin inhibitors, corticosteroids, biological agents, etc.) for antineoplastic chemotherapy in the 3 months preceding the donation;
- Any medication in the 48 h preceding the donation (except contraceptive treatment).

### Randomization

Forty-two of the individuals who respond after 4 weeks of antibiotherapy will be randomized at a ratio of 1:1 to receive either FMT or sham transplantation.

The randomization will be carried out via the Ennov Clinical website: https://nantes-lrsy.hugo-online.fr/CSOnline/ by the study nurse. This randomization process is not stratified by any factors such as age, gender, or center.

However, in addition to the randomization described above, a sero-concordance of CMV, EBV, and *toxoplasma gondii* status between donor and receiver will be required.

### Fecal microbiota or sham transplantation

#### Donor preparation

All healthy donors will be recruited by the University Hospital of Nantes. Donors will be screened in accordance with ANSM recommendations, as described above [38]. The donors’ feces will be collected in a special collection system and always transported on ice. The trial schedule of the donor is showed in Table 4. Processing will be carried out under aerobic conditions. The stools will be weighed to determinate the optimal quantity for the screening tests, safety samples, and transplant preparation. If the stool weight is insufficient (< 70 g), the stool donation will be used for the screening tests and safety samples and the donor will be asked to come back within 15 days for a second stool donation.

**Table 4:** Study schedule for the donor [39]

Study procedures	Screening^a^	Inclusion^a^	1 month Call + or – 14 days
Written Informed Consent	X		
Review of Inclusion / non inclusion Criteria	X	X	
Digestive Status	X	X	
Medical History & Demographics	X		
Physical Exam	X	X	
Vital Signs	X	X	
Blood (safety ANSM)		X	
Stool samples, donation stool (safety ANSM)		X	
Fecalotheque		X	
Concomitant treatment	X	X	
Screening survey	X		
Lightened survey		X	
Safety survey			X

^a^The screening and the inclusion visit could be made at the same time

The preparation will be carried out under the hood: donors’ stools will be mixed in sterile saline without preservatives using a commercial blender to obtain a final concentration of 0.5 g feces/mL. We have therefore opted for a single-use system (containing the saddle and mixing shaft), thus eliminating any risk of cross-contamination.

Materials will be filtered with sterile gauze to obtain a solution without particulate material. The suspension in the container will be prepared using pharmaceutical-grade glycerol as a bacterial cryoprotectant at a final concentration of 10%. The solution will then be placed in 30-mL syringes directly adaptable on the channel of the colonoscope and stored frozen at – 80 °C for a maximum of 365 days [40–44]. All the syringes will be identical and opaque to ensure blinded conditions. The placebo will be sterile saline with pharmaceutical glycerol at a final concentration of 10% in sterile syringes directly adaptable on the channel of the colonoscope.

#### Administration plan

The investigational medicinal product (the Pharmacy will be the only one aware if it is fecal microbiota suspension or placebo) should be administered under the close supervision of an experienced physician and in an environment where full resuscitation facilities are immediately available.

On the day of the transplantation, three 30-mL syringes (two syringes plus a back-up syringe, all either donor or placebo) will be left at room temperature for 2 h to defrost. The contents of the two syringes will be injected 10 cm above the pouch. Finally, a 30-mL syringe of sterile saline will be administered into the pouch to push the fecal solution and avoid waste of FMT.

It should be noted that the randomized individuals will continue antibiotherapy until 48 h before transplantation and will undergo a bowel cleansing (2 L of polyethylene glycol) the night before the FMT and will fast from midnight.

After transplantation, the patient will stay in the left decubitus position for 2 h and then the dorsal decubitus position for 2 h. Four hours after the transplantation, the patient will have a collation; clinical surveillance will continue for 6 h after transplantation to allow vital signs to be monitored (temperature, pulse, blood pressure) every 2 h.

**Fig. 1:**
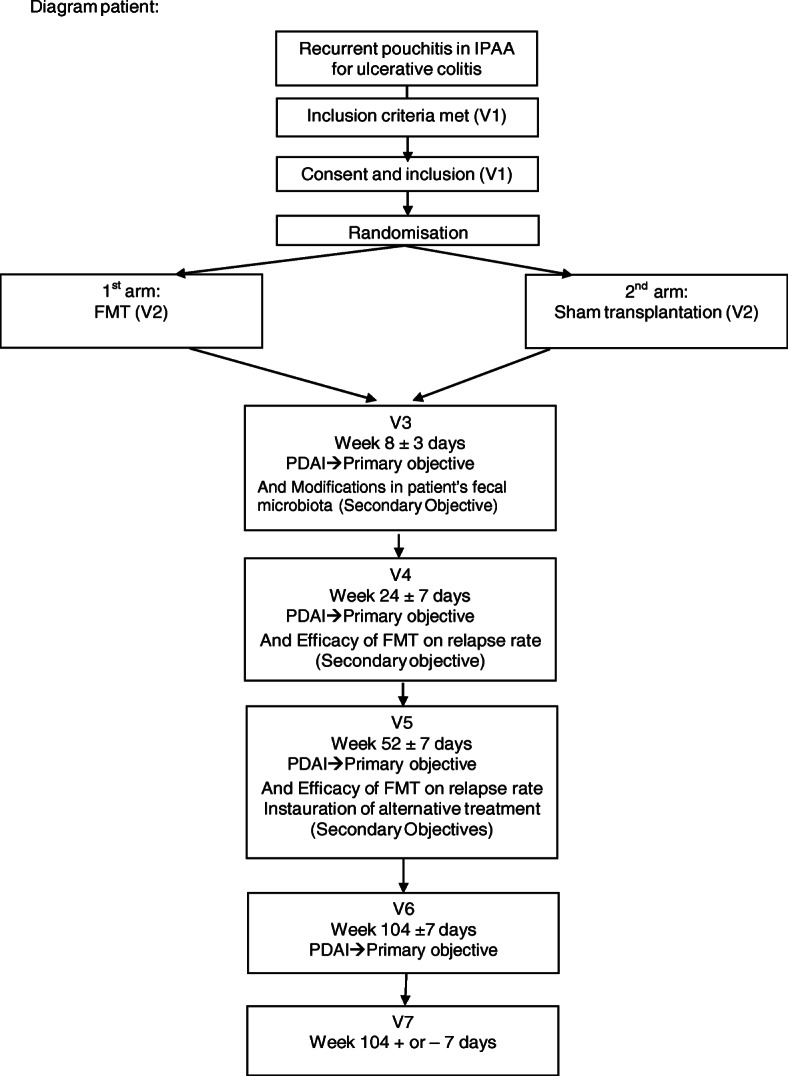
Study diagram

After which, patients will return for visits 2, 6, 12, 18, and 24 months after transplantation, as shown in Fig. 1.

During each visit, physical examination results, adverse events (AEs), and concomitant treatments will be recorded, and Pouchitis Disease Activity Index (PDAI) [45] and IBD Disability Index [46] questionnaires will be filled in. The patient flow chart can be seen in Table 5.

**Table 5:** Study schedule for the patient [39]

Study procedures	V1 Week -2 Screening + or - 3 days	Week -1 Randomisation	V2 Week 0 Transplantation	V3 Week 8 + or - 3 days	V4 Week 24 + or - 7 days	V5 Week 52 + or - 7 days	V6 Week 104 + or - 7 days	Unscheduled Visit (relapse)
Written Informed Consent	X							
Review of Inclusion, non inclusion Criteria	X	X						
Medical History (including UC disease history) & Demographics (including initials, date of birth, sex, tobacco status)	X							
Pouchitis caracteristics and treatments	X							
Physical Exam	X			X	X	X	X	X
PDAI	X			X	X	X	X^a^	X
IBD-Disability Index	X		X	X	X	X	X	X
Flexible rectosigmoïdoscopy (FS)	X		X	X	X	X		X
Biopsies	X (2)^b^		X (4)^c^	X (6)^b+c^	X (6)^b+c^	X (6)^b+c^		X (7)^b+c+d^
Vital Signs	X		X	X	X	X	X	X
Blood samples : blood count, CRP	X^e^		X	X	X	X		X
Biotheque receiver (serum)			X					
Stool Samples (*Clostridium difficile* toxin, parasite)								X
Stool Samples Fecalotheque^f^			X	X	X			X
Prolongation of ATB until 48h prior to the transplantation	X							
Fecal microbiota transplantation/ sham transplantation			X					
Adverse events			X	X	X	X	X	X
Concomittant treatment	X		X	X	X	X	X	X

a: clinical PDAI

b: histology

c: tissutheque

d: CMV

e: Serology will be proceed to check the sero-concorance with the donor (CMV, EBV and toxoplasma gondii)

f: stool samples will be collected before the colonic preparation

Since these visits are not part of routine care, patients will be financially compensated for each visit.

Furthermore, a donor feces biocollection (fecalotheque donor) and a patient biopsy (tissutheque), serum (biotheque) and feces biocollection (fecalotheque receiver) will be centralized at Nantes University Hospital (CHU Nantes) to evaluate the correlation between donor and patient microbiota before and after FMT. The delay of the possible patient’s microbiota resilience after FMT will be constituted. These biocollection samples will be used in future ancillary studies.

## Objectives and statistics

### Objectives

The main objective of the present study is to compare the relapse delay after FMT versus sham transplantation for recurrent pouchitis in IPAA for UC.

The secondary objectives are:

- Efficacy of FMT on the rate of relapse at week 24;
- Efficacy of FMT on the rate of relapse at week 52;
- Instauration of alternative treatment within week 52;
- Safety of FMT in cases of pouchitis;
- Modifications of the fecal microbiota of a patient with ileal pouch for UC complicated by recurrent pouchitis, in remission after antibiotherapy after FMT from a healthy donor at week 8;
- Evolution of health-related disability.

### Endpoints

The primary endpoint is the delay between the date of transplantation and the date of the clinical and endoscopic relapse defined by a PDAI ≥ 7 points.

The PDAI is a 19-point index of pouchitis activity based on both clinical symptoms and endoscopic and histologic findings [45]. Active pouchitis is defined as a PDAI ≥ 7 and remission is defined as a PDAI < 7. Clinical response to treatment can also be quantified by reduction in the PDAI (e.g. a reduction in the PDAI score ≥ 3 from baseline).

The secondary endpoints are:

- Relapse at week 24 defined as a PDAI ≥ 7 at week 24;
- Relapse at week 52 defined as a PDAI ≥ 7 at week 52;
- Delay within the transplantation and the instauration of an antibiotherapy or alternative treatment (immunosuppressive and/or biotherapy and/or corticotherapy);
- Register of AEs defined by Common Terminology Criteria for Adverse Events (4.3) 104 weeks after transplantation according to ANSM recommendation;
- Fecal microbiota engraftment at 8 weeks defined by: Sorensen’s index ([receiver 8 weeks after FMT vs donor] > Sorensen’s index [receiver 8 weeks after FMT vs receiver before FMT]) with Sorensen’s index (receiver 8 weeks after FMT vs donor) = 0.6. To assess this endpoint, fecal microbiota composition will be analyzed for donor sample, receiver sample before FMT and 8 weeks after FMT, using 16S sequencing (Illumina Miseq technology);
- IBD Disability Index at weeks − 5, baseline, and weeks 8, 24, and 52 and unscheduled visit.

### Sample size evaluation

This superiority study is designed to have a statistical power of 80% to detect a significant treatment effect on the first onset of pouchitis. We estimate a survival rate of 25% in the control group versus 60% in the treatment group at 6 months (hazard ratio = 0.3685).

These figures are based on the work on probiotics of Gionchetti and Mimura [18, 19].

A total of 32 events are needed to ensure a statistical power of 80%. For a 48-month enrollment period and 24-month follow-up period, 40 patients are needed to observe 32 events. To ensure this statistical power, a total of 42 patients will be randomized.

This sample size calculation takes into account the interim analysis planned at the first 16 observed events.

### Statistical methods

Individual disposition, demographics, and baseline characteristics will be summarized. Summary statistics for continuous variables will include the number of participants (n), mean, median, standard deviation or standard error, minimum, and maximum. For categorical variables, the frequency and percentage will be given.

It should be noted that if a participant is alive or lost to follow-up without experiencing documented relapse at the time of the analysis, the participant will be censored at the date of last visit.

#### Primary endpoint

The primary endpoint is the relapse delay. This is defined in weeks as the time from transplantation to the date of the clinical and endoscopic relapse. Relapse delay will be estimated using the Kaplan–Meier method. The comparison between treatment groups will be made using the log-rank test.

#### Secondary endpoint

Relapse rates at 24 weeks and 52 weeks will be reported for each treatment group. Delay within the transplantation and the instauration of an antibiotherapy or alternative treatment will be estimated using the Kaplan–Meier method. The comparison between treatment groups will be made using the log-rank test.

#### Disability

Total score on the IBD Disability Index will be estimated using a linear mixed model taking into account score at baseline (week 0), treatment, time (weeks 8 and 24), and treatment by time interaction.

#### Safety analyses

A list of AEs (whether severe or not) will be provided. Descriptive statistics, including the frequency of participants presenting at least one AE and the number of events, will be generated by severity and relationship to treatment. A review of the AEs occurring during the study period will be carried out.

No other additional analyses are planned.

### Management of adverse events

Concerning the study treatment, the systematic review by Wang et al. (“Adverse Events of Fecal Microbiota Transplantation”) [24] will be used as the reference safety information. According to this review, the expected reactions for the lower gastrointestinal route are:

- Gastrointestinal disorders: abdominal pain, abdominal fullness, constipation, bloating, abdominal cramp, diarrhea, nausea, transient relapse of pouchitis, irregularity of bowel movements, increase in stool frequency;
- Infections and infestations: bacteremia, multiorganism bacteremia, urinary tract infection, norovirus gastroenteritis;
- General disorders and administration site conditions: fever, chills;
- Investigations: temporary increase in C-reactive protein (CRP).

Most reactions reported are mild to moderate and concern abdominal pain, abdominal cramping, flatulence, increased stool frequency, constipation, fever, and a temporary increase in CRP.

An increasing intensity or frequency of these expected reactions will be viewed as unexpected. As recommended by ANSM, all medically significant infections will be treated as SUSARs (suspected unexpected serious adverse reactions [SARs]).

It has been suggested that some reactions—such as pouchitis flare, appendicitis, peripheral neuropathy, Sjogren’s disease, idiopathic thrombocytopenic purpura, and rheumatoid arthritis—might be related to FMT. In this trial, however, these events will not be viewed as expected reactions to FMT.

Any AR/AE, whether expected or unexpected, serious or not, will be recorded in real time in the study electronic case report form (eCRF).

All serious AEs/SARs must be reported to the trial’s sponsor if they occur to a research participant:

- From the transplantation;
- As recommended by ANSM (the French regulatory authority), AEs (serious and not serious) must be recorded for 104 weeks after FMT, even if the patient leaves the study (early discontinuation);
- After the end of the patient follow-up period, and without any time limit if the investigator becomes aware of a SAR possibly linked to the experimental treatment.

In case of SAE, a code-break procedure of the pharmacovigilance unit will show immediately the status of the patient.

### Ethical, regulatory, and dissemination aspects

The clinical study will be conducted in accordance with the relevant versions of the French Public Health Code, national and international good clinical practice (GCP) guidelines, and the Declaration of Helsinki, each in the applicable version.

The protocol was written according to a 33-item SPIRIT checklist [39] (see Additional file 1).

It has been possible to carry out the protocol and the trial thanks to an Executive Committee which includes a Scientific Committee and a Steering Committee, created and coordinated by Dr. Trang-Poisson. The Steering Committee follows the development of the trial. It is composed of the members of the Scientific Committee with the addition of the data management team, the study coordinator from the Gastroenterology Department of CHU Nantes who coordinates assistance for patient inclusion in the other centers, and the monitoring Clinical Research Assistant (CRA).

In compliance with French law, the study protocol was submitted to the French regulatory authority (ANSM) and approved on June 8, 2018. It should be noted that in France, the pharmaceutical preparation status requires the preparation of feces for FMT to be carried out under the responsibility of the institution pharmacist. To do this, an application for authorization from a regional regulatory authority must be made. In our case, this authorization was given on May 25, 2018 by the Regional Health Agency of Pays de la Loire.

This clinical study and the donors’ and the patients’ written informed consent was submitted to and approved by the Ethical Review Board of Tours (Comité de Protection des Personnes de Tours, Région centre, Ouest-1) on June 26, 2018. Amendments, if necessary, will be sent to the ethical committee and regulatory authorities for approval. The updated protocol was at version 1.2 on June 8, 2018.

As required, the sponsor has provided an insurance policy to cover the financial consequences of its civil liability in accordance with the regulations.

All the submissions/declarations were made by the Sponsor Department of the University Hospital of Nantes.

The sponsor is responsible also for the quality of the trial data. Data collection for each person participating in the research is realized with an eCRF. The data will be stored directly from the eCRF into the database hosted on a dedicated server, with controlled access (account/password) according to the user role. Any addition, modification, or deletion of data will be recorded in a non-editable electronic file (the audit trail). The principal investigator and all co-investigators undertake to keep the identities of the persons who participate in the study confidential by assigning them a code.

This code will be used for all the eCRF and all the attached documents (reports of imaging exams, biology, etc.). It will be the only information which will make it possible to make the connection with the patient retrospectively.

The coding rule is the following: first letter of first name + first letter of surname +/– month and year of birth and the randomization number.

The data collected during the study will be processed electronically in accordance with the requirements of the CNIL, the French Data Protection Authority (in compliance with the French Reference Methodology MR001). The CNIL is an independent French administrative regulatory body whose mission is to ensure that data privacy law is applied to the collection, storage, and use of personal data.

An inspection or audit may take place as part of this study, performed by the sponsor and/or by the regulatory authorities. Inspectors will check the documents, logistics, records, and any other resources that the authorities consider to be associated with the clinical trial and that may be located at the trial site itself.

The trial results will be published in international gastroenterological, medical, and scientific journals and presented in national and international congresses. The datasets analyzed during the current study will be available from the corresponding author on reasonable request. The investigators, who will share only with each other the final trial dataset, will follow the rules and guidelines of the International Committee for Medical Journal Editors (ICMJE) [47]. The trial’s sponsor and the French Ministry of Health, who provided the grant, have to be cited in the publication.

## Discussion

This is one of the first randomized studies on the use of FMT in the prevention of chronic antibiotic-dependent pouchitis. Moreover, the aim of the present study is to promote withdrawal from antibiotics and avoid immunosuppressive treatments and there is a lack of clinical trials available on this topic.

Indeed, although the number of studies on FMT and UC that are of high methodological quality has increased of late, the optimal conditions for durable FMT engraftment and maximal remission remain unclear. Moreover, there is also a need for high-quality, placebo-controlled trials evaluating the safety and efficacy of established treatments for pouchitis. Indeed, a recent Cochrane review concluded that only VSL#3-type probiotics were statistically superior to placebos in the prevention or treatment of pouchitis [16, 25]. Given the demand for new therapies and lack of evidence for existing treatments, it is of paramount importance that trial design for pouchitis be efficient [14].

As has been previously mentioned, though the majority of initial cases of pouchitis are easily managed with a short course of antibiotics, in around 5%–15% of cases, inflammation of the pouch becomes chronic with very few treatments available. In addition to this clinical problem for the patient, long-term antibiotics are not a solution. Indeed, in April 2014, a World Health Organization report provided the most comprehensive picture of antibiotic resistance to date [48].

If the present study proves to be of interest, the centralized procedure for donor selection and fecal conservation will need to be set up. Under this protocol, in addition to the admission of patients having undergone an IPAA for UC, and while waiting to know if they respond to antibiotic therapy, the donation of feces by healthy volunteers must be managed in accordance with ANSM guidelines including routine tests for ESBL-producing organisms in donor-screening protocol since march 2014 [38].

### Trial status

The updated protocol was at version 1.2 on June 8, 2018.

The first donor gave their informed consent on December 5, 2018, but did not qualify for donation.

Currently, no patients have been included.

The inclusion period for donors and patients is 48 months, with recruitment scheduled to end in February 2024. The total duration of the study is 76 months, with an anticipated end date of June 2026.

## Supplementary information

**Additional file 1.** PoCa ‘s SPIRIT checklist [39].

## Data Availability

Data sharing is not applicable to this article as no datasets were generated or analyzed during the current study. The data from the completed trial will not be shared and will only be transmitted to the sponsor. Data collected during the test may be processed electronically, in accordance with the requirements of the CNIL (compliance with reference methodology MR001).

## Abbreviations

CD: Crohn’s disease
CDI: *Clostridium difficile* infection
CMV: Cytomegalovirus
CRA: Clinical Research Assistant
CRP: C-reactive protein
EBV: Epstein–Barr virus
eCRF: Electronic case report form
ESBC: Extended spectrum β-lactamase
FDA: Food and Drug Administration
FMT: Fecal microbiota transplantation
IBD: Inflammatory bowel disease
ICF: Informed consent Form
IPAA: Ileal pouch–anal anastomosis
PDAI: Pouchitis Disease Activity Index
RCT: Randomized clinical trial
UC: Ulcerative colitis
