# Supplementary material for: Impact of fecal microbiota transplantation on chronic recurrent pouchitis in ulcerative colitis with ileo-anal anastomosis: study protocol for a prospective, multicenter, double-blind, randomized, controlled trial
Source: Trials. 2020 Jun 3;21:455. doi: 10.1186/s13063-020-04330-1 (PMC7267479; doi:10.1186/s13063-020-04330-1)
Supplement: Supplementary file 1 — Additional file 1. PoCa ‘s SPIRIT checklist [39]. [file 13063_2020_4330_MOESM1_ESM.doc]

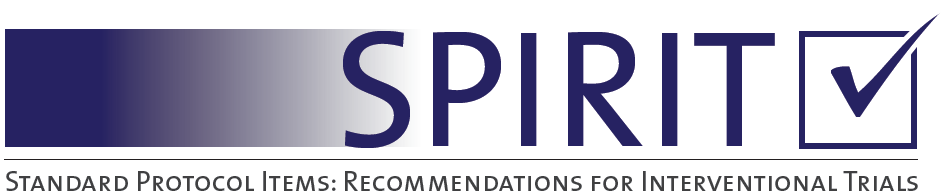


SPIRIT 2013 Checklist: Recommended items to address in a clinical trial protocol and related documents*

| Section/item | ItemNo | Description |
| --- | --- | --- |
| **Administrative information** | | |
| Title | 1 | Descriptive title identifying the study design, population, interventions, and, if applicable, trial acronym **Pg 1** in Title |
| Trial registration | 2a | Trial identifier and registry name. If not yet registered, name of intended registry **Pg 2** in Abstract – Trial registration |
|  | 2b | All items from the World Health Organization Trial Registration Data Set  **Item 1** 🡺 **Pg1** Abstract in Trial registration and **Pg 22** Trial Status  **Item 2** 🡺 Ditto item 1  **Item 3** 🡺 *Not Applicable*  **Item 4** 🡺 **Pg 24** in Funding  **Item 5** 🡺 **Pg 20** in Ethical, Regulatory and dissemination aspect  **Item 6** 🡺*Not Applicable*  **Item 7** 🡺 **Pg 1** Authors from Sponsor Department of Nantes University Hospital  **Item 8** 🡺 **Pg 1** corresponding Author  **Item 9** 🡺 **Pg 24-25** in Tittle of the trial in Funding  **Item 10** 🡺 Ditto item 9  **Item 11** 🡺 **Pg 11** in Methods/Design – Study Design  Item 12 🡺 **Pg 5- 10** Background and **Pg 11** in Methods/Design – Study population – patient  **Item 13** 🡺 **Pg 13-15** in Methods/Design –Study schedule - Fecal Microbiota or sham transplantation  **Item 14** 🡺see Box 1 to 3 and **Pg 11-12** in Methods/Design - Study Population  **Item 15** 🡺 **Pg 11** in Methods/Design – Study design  **Item 16** 🡺 **Pg 22** in Trial Status  **Item 17** 🡺 **Pg 17** in STATISTIC – Sample size evaluation  **Item 18** 🡺 Ditto item 16  **Item 19** 🡺 **Pg 16** in OBJECTIVES AND STATISTICS – Endpoints  **Item 20** 🡺 Ditto item 19  **Item 21** 🡺 **Pg 19-20** in ETHICAL,REGULATORY AND DISSEMINATION ASPECTS  **Item 22** 🡺 **Pg 22** in Trial Status  **Item 23** 🡺 Not Applicable  **Item 24** 🡺 **Pg 19-20** in Ethical, Regulatory and dissemination aspect |
| Protocol version | 3 | Date and version identifier **Pg 22** Trial Status |
| Funding | 4 | Sources and types of financial, material, and other support **Pg 21** in Funding |
| Roles and responsibilities | 5a | Names, affiliations, and roles of protocol contributors  **Pg 1** in the first page of the manuscript; **pg 11** in METHODS/DESIGN study population, **pg 25** in Author’s contribution |
|  | 5b | Name and contact information for the trial sponsor **Pg 1** Authors from Sponsor Department of Nantes University Hospital and **Pg 19-20** in Ethical, Regulatory and dissemination aspect |
|  | 5c | Role of study sponsor and funders, if any, in study design; collection, management, analysis, and interpretation of data; writing of the report; and the decision to submit the report for publication, including whether they will have ultimate authority over any of these activities **Pg 19-20** in Ethical, Regulatory and dissemination aspecT |
|  | 5d | Composition, roles, and responsibilities of the coordinating centre, steering committee, endpoint adjudication committee, data management team, and other individuals or groups overseeing the trial, if applicable (see Item 21a for data monitoring committee) **Pg 19-20** in Ethical, Regulatory and dissemination aspecT |
| Introduction |  |  |
| Background and rationale | 6a | Description of research question and justification for undertaking the trial, including summary of relevant studies (published and unpublished) examining benefits and harms for each intervention **Pg 5 - 10**Background |
|  | 6b | Explanation for choice of comparators **Pg 5-10** Background |
| Objectives | 7 | Specific objectives or hypotheses **Pg 15** in OBJECTIVES AND STATISTICS - Objectives |
| Trial design | 8 | Description of trial design including type of trial (eg, parallel group, crossover, factorial, single group), allocation ratio, and framework (eg, superiority, equivalence, noninferiority, exploratory) **Pg 11** in METHODS/DESIGN – Study Design, **Pg 17** OBJECTIVES AND STATISTICS – Sample size evaluation and **Pg 17** STATISTIC - Statistical methods |
| Methods: Participants, interventions, and outcomes | | |
| Study setting | 9 | Description of study settings (eg, community clinic, academic hospital) and list of countries where data will be collected. Reference to where list of study sites can be obtained **Pg 11-12** in METHODS/DESIGN – Study Design and Study Population |
| Eligibility criteria | 10 | Inclusion and exclusion criteria for participants. If applicable, eligibility criteria for study centres and individuals who will perform the interventions (eg, surgeons, psychotherapists) see Box 1 to 3 and **Pg 11 - 13** in Methods/Design - Study Population and Study schedule |
| Interventions | 11a | Interventions for each group with sufficient detail to allow replication, including how and when they will be administered **Pg 13 - 15** in Methods/Design - Fecal Microbiota or sham transplantation and administration plan |
|  | 11b | Criteria for discontinuing or modifying allocated interventions for a given trial participant (eg, drug dose change in response to harms, participant request, or improving/worsening disease) Not applicable |
|  | 11c | Strategies to improve adherence to intervention protocols, and any procedures for monitoring adherence (eg, drug tablet return, laboratory tests) administered **Pg 14** in Methods/Design - Fecal Microbiota or sham transplantation - administration plan |
|  | 11d | Relevant concomitant care and interventions that are permitted or prohibited during the trial **Pg 11 - 12**  in Methods/Design – Study PopuLaTION |
| Outcomes | 12 | Primary, secondary, and other outcomes, including the specific measurement variable (eg, systolic blood pressure), analysis metric (eg, change from baseline, final value, time to event), method of aggregation (eg, median, proportion), and time point for each outcome. Explanation of the clinical relevance of chosen efficacy and harm outcomes is strongly recommended **Pg 16** in Methods/Design – OBJECTIVES AND STATISTICS- Endpoints |
| Participant timeline | 13 | Time schedule of enrolment, interventions (including any run-ins and washouts), assessments, and visits for participants. A schematic diagram is highly recommended (see Figure) Figure 1 and Figure 2 |
| Sample size | 14 | Estimated number of participants needed to achieve study objectives and how it was determined, including clinical and statistical assumptions supporting any sample size calculations **Pg 17** in Methods/Design – OBJECTIVES AND STATISTICS – Sample size evaluation |
| Recruitment | 15 | Strategies for achieving adequate participant enrolment to reach target sample size **Pg 11-12** in Methods/Design – Study PopuLaTION |
| **Methods: Assignment of interventions (for controlled trials)** | | |
| Allocation: |  |  |
| Sequence generation | 16a | Method of generating the allocation sequence (eg, computer-generated random numbers), and list of any factors for stratification. To reduce predictability of a random sequence, details of any planned restriction (eg, blocking) should be provided in a separate document that is unavailable to those who enrol participants or assign interventions **Pg 13** in Methods/Design – Study SCHEDULE - Randomization |
| Allocation concealment mechanism | 16b | Mechanism of implementing the allocation sequence (eg, central telephone; sequentially numbered, opaque, sealed envelopes), describing any steps to conceal the sequence until interventions are assigned ditto item 16a |
| Implementation | 16c | Who will generate the allocation sequence, who will enrol participants, and who will assign participants to interventions ditto item 16a |
| Blinding (masking) | 17a | Who will be blinded after assignment to interventions (eg, trial participants, care providers, outcome assessors, data analysts), and how **Pg 14** in Methods/Design – Study Schedule –  fecal microbiota or sham transplantation - Administration plan |
|  | 17b | If blinded, circumstances under which unblinding is permissible, and procedure for revealing a participant’s allocated intervention during the trial – pg19 in ADVERSE EVENT MANAGEMENT |
| **Methods: Data collection, management, and analysis** | | |
| Data collection methods | 18a | Plans for assessment and collection of outcome, baseline, and other trial data, including any related processes to promote data quality (eg, duplicate measurements, training of assessors) and a description of study instruments (eg, questionnaires, laboratory tests) along with their reliability and validity, if known. Reference to where data collection forms can be found, if not in the protocol see Figure 2 and **Pg 14** in Methods/Design – Study Schedule – Administration plan |
|  | 18b | Plans to promote participant retention and complete follow-up, including list of any outcome data to be collected for participants who discontinue or deviate from intervention protocols **Pg 14** in Methods/Design – Study Schedule – Administration plan |
| Data management | 19 | Plans for data entry, coding, security, and storage, including any related processes to promote data quality (eg, double data entry; range checks for data values). Reference to where details of data management procedures can be found, if not in the protocol see in the Protocol |
| Statistical methods | 20a | Statistical methods for analysing primary and secondary outcomes. Reference to where other details of the statistical analysis plan can be found, if not in the protocol. **Pg 17-18** in OBJECTIVES AND STATISTICS – Statistical methods |
|  | 20b | Methods for any additional analyses (eg, subgroup and adjusted analyses) ditto item 20a |
|  | 20c | Definition of analysis population relating to protocol non-adherence (eg, as randomised analysis), and any statistical methods to handle missing data (eg, multiple imputation) ditto item 20c |
| **Methods: Monitoring** | | |
| Data monitoring | 21a | Composition of data monitoring committee (DMC); summary of its role and reporting structure; statement of whether it is independent from the sponsor and competing interests; and reference to where further details about its charter can be found, if not in the protocol. Alternatively, an explanation of why a DMC is not needed As FMT is not a first application on humans; a DMC was not required by the regulatory authorities. |
|  | 21b | Description of any interim analyses and stopping guidelines, including who will have access to these interim results and make the final decision to terminate the trial **Pg 17** in OBJECTIVES AND STATISTICS – Sample size evaluation |
| Harms | 22 | Plans for collecting, assessing, reporting, and managing solicited and spontaneously reported adverse events and other unintended effects of trial interventions or trial conduct **Pg 18** Adverse event management |
| Auditing | 23 | Frequency and procedures for auditing trial conduct, if any, and whether the process will be independent from investigators and the sponsor see in Protocol |
| Ethics and dissemination | | |
| Research ethics approval | 24 | Plans for seeking research ethics committee/institutional review board (REC/IRB) approval **Pg 19-20** in Ethical, Regulatory and dissemination aspect |
| Protocol amendments | 25 | Plans for communicating important protocol modifications (eg, changes to eligibility criteria, outcomes, analyses) to relevant parties (eg, investigators, REC/IRBs, trial participants, trial registries, journals, regulators) approval **Pg 19-20** in Ethical, Regulatory and dissemination aspect |
| Consent or assent | 26a | Who will obtain informed consent or assent from potential trial participants or authorised surrogates, and how (see Item 32) **Pg 11** in Methods/Design – Study PopuLaTION |
|  | 26b | Additional consent provisions for collection and use of participant data and biological specimens in ancillary studies, if applicable Pg15 in Methods/Design – Study SCHEDULE |
| Confidentiality | 27 | How personal information about potential and enrolled participants will be collected, shared, and maintained in order to protect confidentiality before, during, and after the trial see in protocole |
| Declaration of interests | 28 | Financial and other competing interests for principal investigators for the overall trial and each study site COMPETITING INTEREST pg 24 |
| Access to data | 29 | Statement of who will have access to the final trial dataset, and disclosure of contractual agreements that limit such access for investigators see in protocole |
| Ancillary and post-trial care | 30 | Provisions, if any, for ancillary and post-trial care, and for compensation to those who suffer harm from trial participation pg20 in Ethical, Regulatory and dissemination aspect |
| Dissemination policy | 31a | Plans for investigators and sponsor to communicate trial results to participants, healthcare professionals, the public, and other relevant groups (eg, via publication, reporting in results databases, or other data sharing arrangements), including any publication restrictions **Pg 19 - 20** in Ethical, Regulatory and dissemination aspect |
|  | 31b | Authorship eligibility guidelines and any intended use of professional writers **Pg 19 - 20** in Ethical, Regulatory and dissemination aspECT |
|  | 31c | Plans, if any, for granting public access to the full protocol, participant-level dataset, and statistical code Not applicable |
| Appendices |  |  |
| Informed consent materials | 32 | Model consent form and other related documentation given to participants and authorised surrogates see in protocol |
| Biological specimens | 33 | Plans for collection, laboratory evaluation, and storage of biological specimens for genetic or molecular analysis in the current trial and for future use in ancillary studies, if applicable Pg15 in Methods/Design – Study SCHEDULE |

*It is strongly recommended that this checklist be read in conjunction with the SPIRIT 2013 Explanation & Elaboration for important clarification on the items. Amendments to the protocol should be tracked and dated. The SPIRIT checklist is copyrighted by the SPIRIT Group under the Creative Commons “[Attribution-NonCommercial-NoDerivs 3.0 Unported](http://www.creativecommons.org/licenses/by-nc-nd/3.0/)” license.
